# Vorinostat and metformin sensitize EGFR-TKI resistant NSCLC cells via BIM-dependent apoptosis induction

**DOI:** 10.18632/oncotarget.21225

**Published:** 2017-09-23

**Authors:** Hengyi Chen, Yubo Wang, Caiyu Lin, Conghua Lu, Rui Han, Lin Jiao, Li Li, Yong He

**Affiliations:** ^1^ Department of Respiratory Disease, Daping Hospital, Third Military Medical University, Chongqing, China

**Keywords:** vorinostat, metformin, BIM, apoptosis, EGFR-TKI resistance

## Abstract

There is a close relationship between low expression of BIM and resistance to epidermal growth factor receptor tyrosine kinase inhibitor (EGFR-TKI). Vorinostat is a pan-histone deacetylase inhibitor (HDACi) that augments BIM expression in various types of tumor cells, however, this effect is attenuated by the high expression of anti-apoptotic proteins in EGFR-TKI resistant non-small cell lung cancer (NSCLC) cells. Vorinostat in combination with metformin – a compound that can inhibit anti-apoptotic proteins expression, might cooperate to activate apoptotic signaling and overcome EGFR-TKI resistance. This study aimed to investigate the cooperative effect and evaluate possible molecular mechanisms. The results showed that vorinostat combined with gefitinib augmented BIM expression and increased the sensitivity of EGFR-TKI resistant NSCLC cells to gefitinib, adding metformin simultaneously could obviously inhibit the expression of anti-apoptotic proteins, and further increased expression levels of BIM and BAX, and as a result, further improved the sensitivity of gefitinib both on the NSCLC cells with intrinsic and acquired resistance to EGFR-TKI. In addition, autophagy induced by gefitinib and vorinostat could be significantly suppressed by metformin, which might also contribute to enhance apoptosis and improve sensitivity of gefitinib. These results suggested that the combination of vorinostat and metformin might represent a novel strategy to overcome EGFR-TKI resistance associated with BIM-dependent apoptosis in larger heterogeneous populations.

## INTRODUCTION

Despite epidermal growth factor receptor tyrosine kinase inhibitor (EGFR-TKI) have shown dramatic therapeutic efficacy, resistance is almost inevitable and is considered a major clinical problem [1]. 3rd generation EGFR-TKIs represented by osimertinib, exhibited powerful effects against T790 M resistance mutation [2, 3], but it is also inevitably challenged by the problem of acquired resistance [4, 5]. Nowadays, 1st generation EGFR-TKIs still are standard treatments for advanced non-small-cell lung cancer (NSCLC) patients. Accordingly, further research on the combination strategies for overcoming EGFR-TKI resistance has an important clinical significance and applicable value.

Previous research found that the pro-apoptotic signaling BH3-only protein BIM was significantly associated with the clinical effects of EGFR-TKI [6]. Low to intermediate BIM expression levels [6] are closely associated with EGFR-TKI resistance by several mechanisms including the secondary mutation T790M [7], Met amplification [4], activation of the PI3K/AKT/mTOR signaling pathway, and others [8]. Thus, it seems plausible that overcoming resistance to EGFR-TKI by a variety of mechanisms might be a straightforward approach that is dependent on enhancing expression of BIM [9].

Research suggests that vorinostat, which is a pan-histone deacetylase inhibitor (HDACi), can epigenetically restore BIM function and sensitivity of NSCLC cells expressing the BIM deletion polymorphism to EGFR-TKI [10]. Vorinostat is considered the optimal treatment of choice in the clinic when used in combination with chemotherapeutic or molecular targeted therapy, due in large part to the transcriptional activation of BIM [11, 12] and manageable clinical side-effects. However, the combined therapy of HDACi and erlotinib exhibit limited efficacy in unselected patients with advanced NSCLC [13]. A possible reason for this lie in the knowledge that HDACi-induced apoptosis is attenuated by the B-cell lymphoma 2 (Bcl-2) family of anti-apoptotic proteins, including Bcl-xL and Mcl-1 [14], which have been shown to be up-regulated in EGFR-TKI resistant NSCLC cells [15, 16]. Moreover, HDACi upregulates BIM and enhances BIM/Bcl-2 binding, which potently attenuates apoptosis [17]. In addition, autophagy induced by HDACi or EGFR-TKI, may switch from a pro-apoptotic function to a pro-survival signal that drives acquired resistance [17–19]. Thus, vorinostat combined with drugs that are capable of inhibiting expression of anti-apoptotic proteins might represent a promising strategy to enhance EGFR-TKI sensitivity and overcoming EGFR-TKI resistance.

The hypoglycemic drug metformin displays adjuvant anti-cancer effects [20]. Our previous studies also showed that metformin could enhance EGFR-TKI sensitivity in EGFR-TKI resistant NSCLC cells [21], metformin use was associated with improved survival and delayed onset of acquired resistance to EGFR-TKI in patients with NSCLC and diabetes mellitus type 2 [22]. Remarkably, it was previously reported that metformin could inhibit the expression of anti-apoptotic proteins such as Bcl-2 and Mcl-1 [23], thus it might be able to increase HDACi-induced apoptosis. Additionally, metformin could directly increase BIM expression by blocking the AKT signaling pathway [21, 24]. However, metformin does not seem to be sufficient to treat cancer alone, which increases the need for this agent to be combined with other drugs with the intent of revealing its synergistic or cooperative cytotoxic effect [25].

Taken together, metformin and vorinostat may cooperate to target the BIM signaling pathway to overcome TKI resistance with super-compound effects. The current research aims to study the effect and mechanisms involved by the combined use of metformin and vorinostat on TKI resistance. It is intended that such research will demonstrate complementary synergy of cotreatment with metformin and vorinostat on TKI sensitivity, thus providing a new therapeutic approach that could be generally adopted to treat NSCLC patients with EGFR mutations.

## RESULTS

### Metformin combined with vorinostat synergistically augmented gefitinib-induced apoptosis in EGFR-TKI resistant NSCLC cells

**Figure 1:**
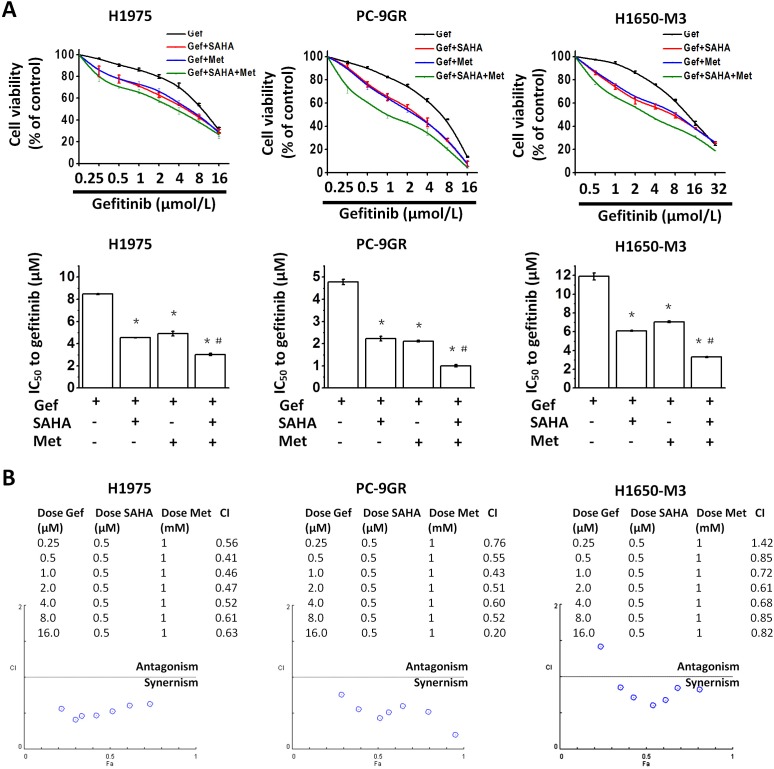
Metformin and vorinostat synergistically enhanced gefitinib-induced cytotoxicity in EGFR-TKI resistant NSCLC cell-lines **(A)** Vorinostat and/or metformin increased the sensitivity of EGFR-TKI-resistant cells to gefitinib. Cell viabilities of H1975, PC-9GR and H1650-M3 cells that were treated with the indicated doses of gefitinib for 48 hr were assessed by the MTT assay. ^*^p < 0.05 as compared with gefitinib treatment alone; ^#^*p* < 0.05 as compared with gefitinib combined with vorinostat or metformin. **(B)** The combination index (CI) values for the combined treatment with gefitinib, vorinostat and metformin were calculated using the CompnSyn software tool (Paramus, NJ, USA). Data presented were representative for at least three independent experiments. Gef: gefitinib; SAHA: vorinostat; Met: metformin.

To evaluate the interaction of gefitinib with vorinostat and metformin, H1975, PC-9GR (Supplementary Figure 1) and H1650-M3 cells were treated with a fixed dose combination (i.e., vorinostat, 0.5μM, metformin, 1mM, and increasing concentrations of gefitinib) for 48 hr, following which, growth inhibition was measured by the MTT viability assay. As compared with gefitinib alone or gefitinib in combination with vorinostat (0.5μM) or metformin (1 mM), all types of cells treated with the fixed dose combination (i.e., gefitinib plus vorinostat and metformin), exhibited a clearly decreased viability (Figure 1A).

The combination index (CI) values were all<1 with the exception of the combination of metformin (1 mM), vorinostat (0.5μM) and gefitinib (0.25μM) in H1650-M3 cells (Figure 1B), which indicated that there was a synergistic interaction between vorinostat, metformin and gefitinib. Additionally, it was found that 48 hr culture in vorinostat (0.5μM) and metformin (1 mM) containing medium could further potentiate the effects of gefitinib on the proliferation and invasiveness of H1975 (Supplementary Figure 2A, 2B) and PC-9GR cells (Supplementary Figure 3A, 3B). Of note, vorinostat (0.5μM) and metformin (1 mM) alone only slightly decreased cell viability in cells used in the study (Supplementary Figure 4A-4C).

**Figure 2:**
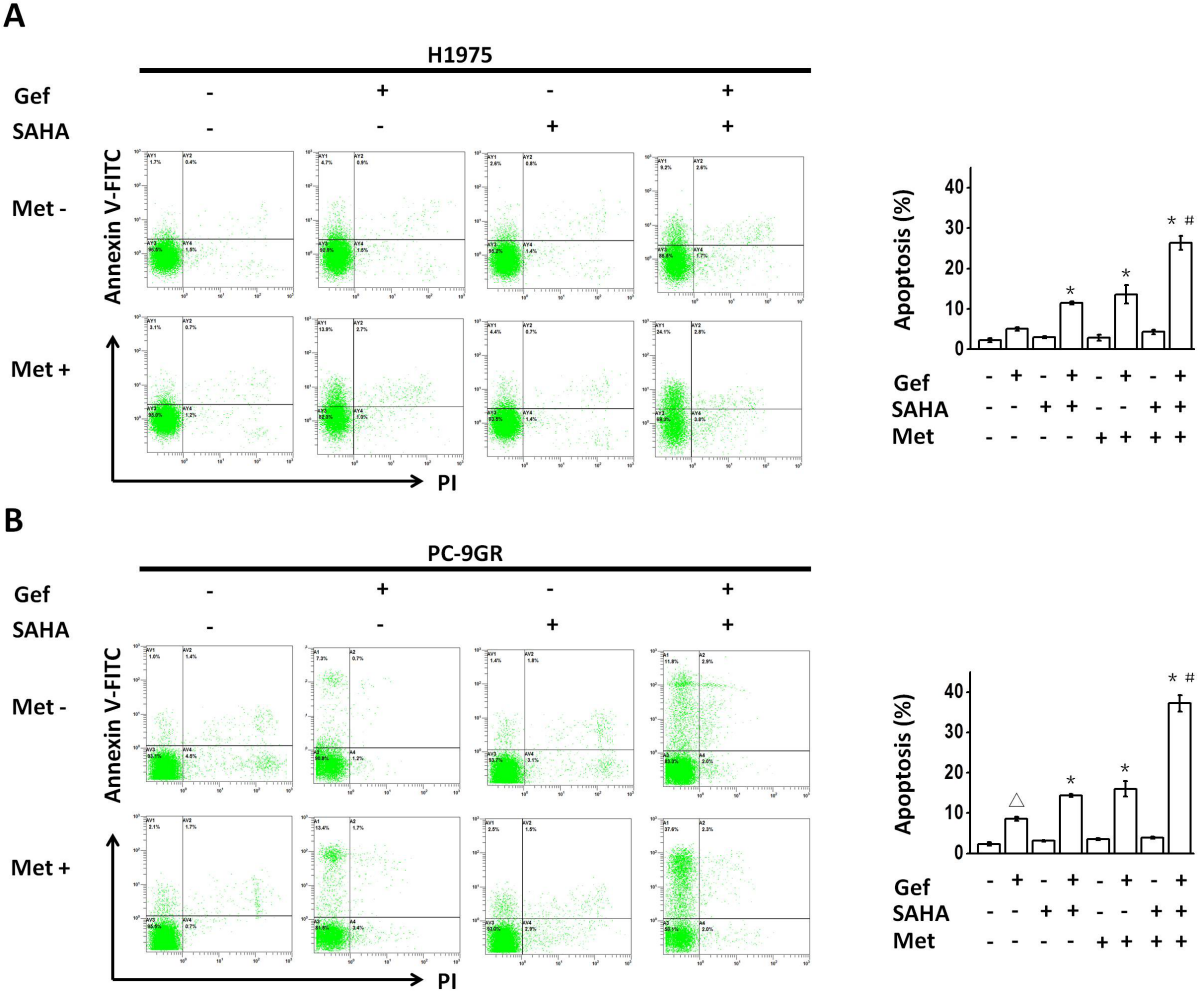
(**A** and **B**) Metformin and vorinostat showed synergistic apoptosis that was promoted with gefitinib in EGFR-TKI-resistant NSCLC cell-lines. Flow cytometric analysis by Annexin V–FITC/PI staining detected apoptosis induction. Images were representative of three independent experiments. ^*^p < 0.05 as compared with gefitinib treatment alone, ^#^*p* < 0.05 as compared with gefitinib combined with vorinostat or metformin, Δ*p* < 0.05 as compared with the control (untreated) cells. Gef: gefitinib; SAHA: vorinostat; Met: metformin.

We next performed flow cytometric analysis to determine the apoptotic promoting effect following the combined use of vorinostat and metformin in NSCLC cells with intrinsic or acquired resistance to gefitinib. Although vorinostat (0.5 μM for 48hr) or metformin (1mM for 48hr) treatment alone had little effect on the apoptosis of either H1975 or PC-9GR cells, vorinostat or metformin significantly increased the apoptosis that was induced by gefitinib in H1975 and PC-9GR cells, and combined use of vorinostat and metformin further augmented this effect (Figure 2A and 2B).

### Metformin combined with vorinostat and gefitinib regulates the apoptosis signaling pathway in EGFR-TKI resistant NSCLC cells

**Figure 3:**
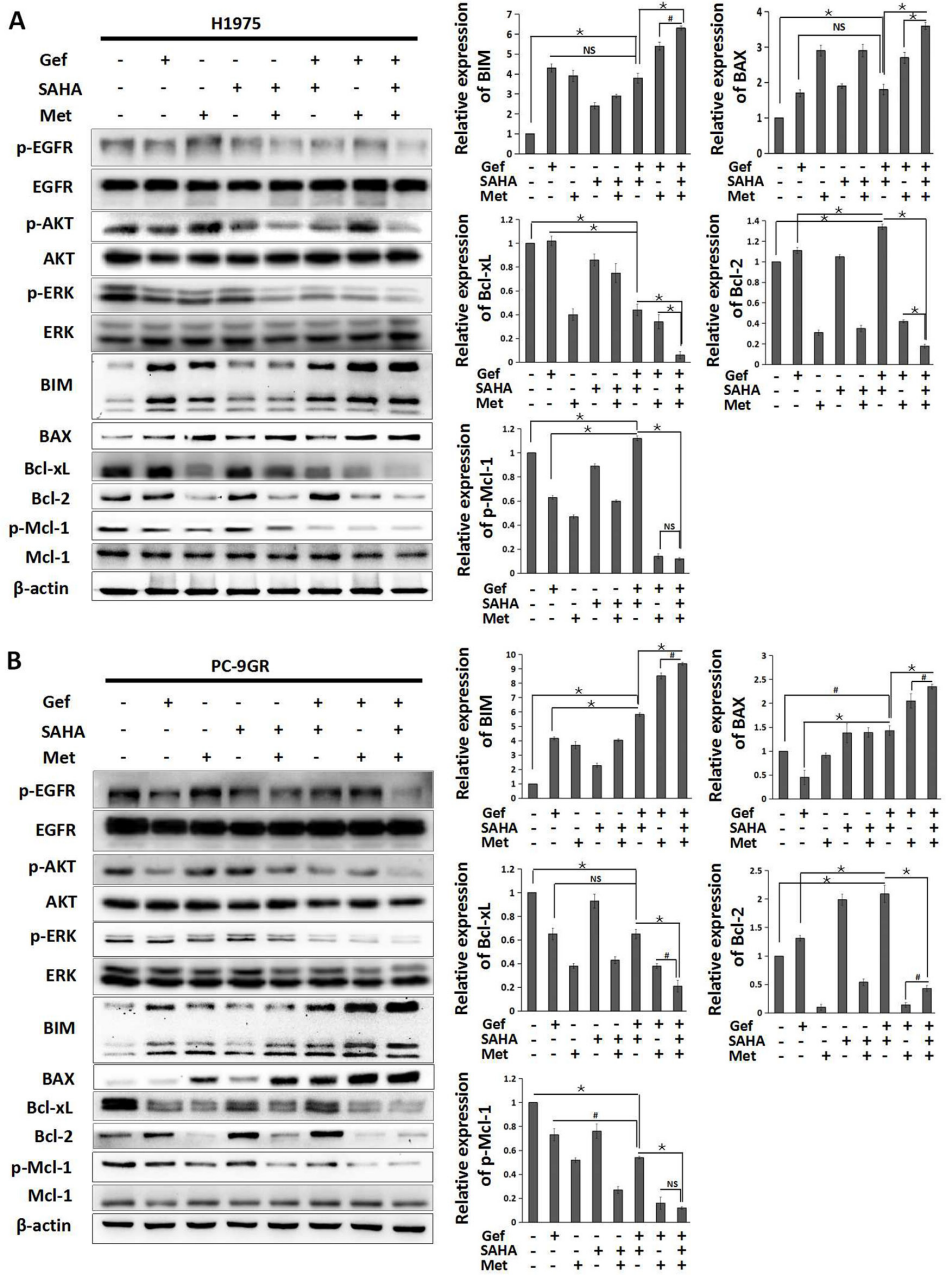
Metformin in combination with vorinostat and gefitinib regulates the apoptosis signaling pathway in EGFR-TKI resistant NSCLC cells **(A)** H1975 and **(B)** PC-9GR cells were treated with vorinostat (0.5 μM) and/or gefitinib (IC25) with/without metformin (1 mM) for 48 hr. Whole cell protein lysates were immune-blotted with the indicated antibodies. Relative expression of BIM, BAX, Bcl-xL, Bcl-2 and p-Mcl-1 were expressed as the mean±standard from three independent experiments. ^*^p < 0.001; ^#^*p* < 0.05; NS: not significant (Student's t test). Gef: gefitinib; SAHA: vorinostat; Met: metformin.

To identify the molecular mechanisms responsible for overcoming intrinsic and acquired TKI resistance to vorinostat and metformin, we examined the effects of vorinostat and/or metformin on the apoptosis signaling pathway. By Western blot analysis, the results showed that the combined treatment of gefitinib and vorinostat in H1975 cells decreased the expression of Bcl-xL and p-Mcl-1, whereas with no effect on BIM and BAX expression when compared with gefitinib alone. In PC-9GR and H1650-M3 cells, combined use gefitinib and vorinostat downregulated the expression of p-Mcl-1 and upregulated the expression of BIM and BAX, with no effect on Bcl-xL. What’more, gefitinib in combination with vorinostat could upregulate the expression of Bcl-2 in all cell lines. Importantly, adding metformin simultaneously could obviously inhibit the expressions of anti-apoptotic proteins Bcl-2, Bcl-xL and p-Mcl-1, and further increased expression levels of BIM and BAX (Figure 3A and 3B, Supplementary Figure 5).

Since studies showed that the induction of BIM was a consequence of both post-translational modification and transcriptional induction that depend on ERK and the AKT pathway inhibition [26, 27], we also found that this combination significantly diminished phosphorylation of ERK and AKT, and inhibited the phosphorylation of EGFR (Figure 3A and 3B, Supplementary Figure 5). These data demonstrated that combined use of vorinostat, metformin and gefitinib could significantly activate the apoptosis pathway in EGFR-TKI resistant NSCLC cells.

### BIM had a significant functional role for the synergetic effect of vorinostat and metformin

**Figure 4:**
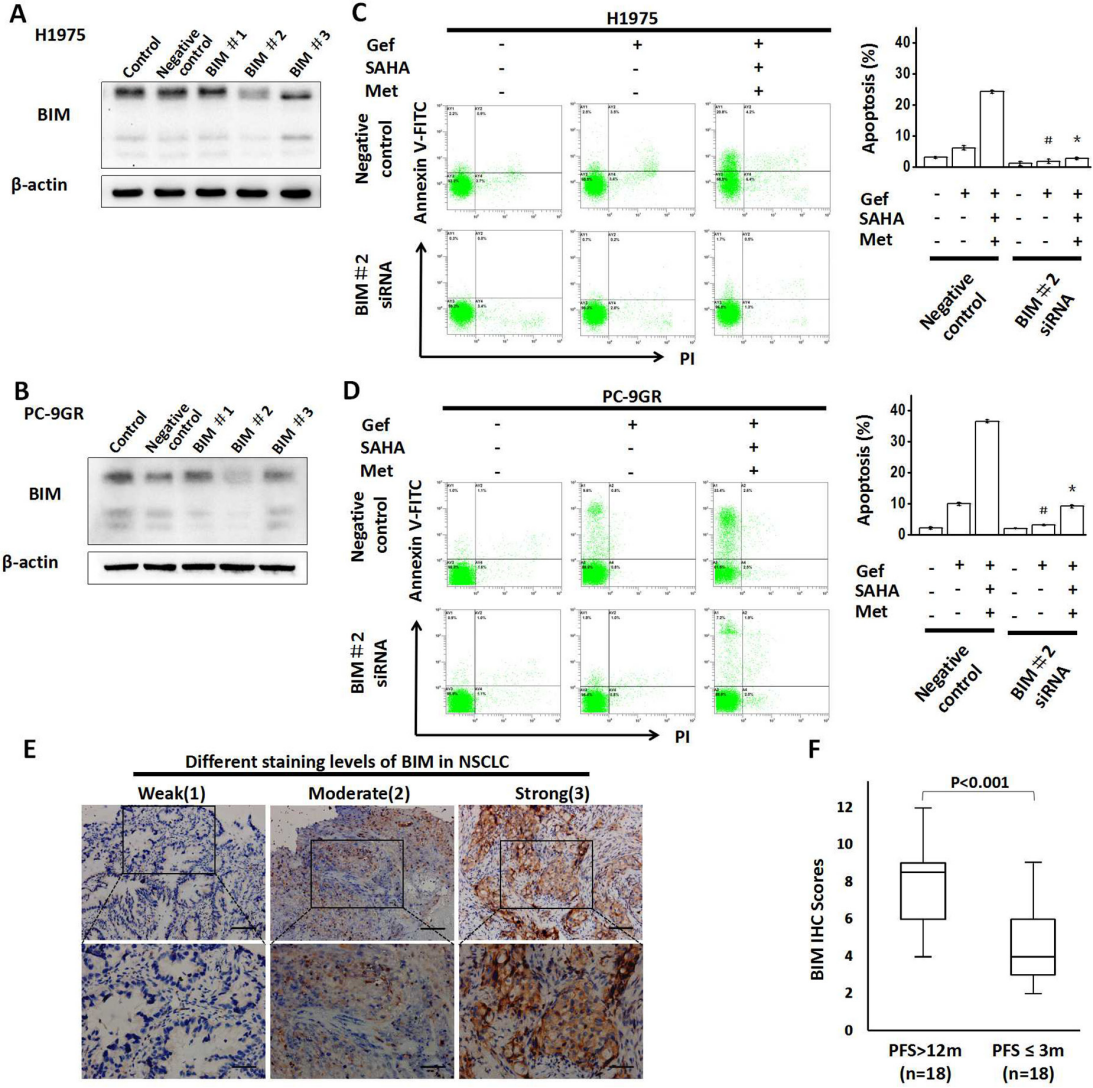
Knockdown of BIM expression attenuates apoptosis **(A)** H1975 and **(B)** PC-9GR cells were transfected with BIM siRNA or control oligo-nucleotides for 48 hr respectively. Lysates were then collected and proteins were analyzed by immunoblotting. **(C)** H1975 and **(D)** PC-9GR cells were transfected with siRNAs that targeted BIM 48 hr prior to drug treatment (gefitinib alone or combined with vorinostat and metformin) for 48 hr, and then apoptotic cells were enumerated by flow cytometric analysis by Annexin V–FITC/PI staining. ^*^p < 0.05 as compared with the negative control treated with the three-drug regimen; ^#^*p* < 0.05 as compared with the negative control that was treated with gefitinib alone. **(E)** Immunohistochemistry (IHC) assays of BIM expression in the tumor tissues of NSCLC patients displaying the EGFR-mutant. The staining intensity in NSCLC was scored from 1-3. (The full lines in upper and lower panels represented 200 and 100μm, respectively). **(F)** Comparison of BIM expression by PFS. Gef: gefitinib; SAHA: vorinostat; Met: metformin; PFS: progression-free survival.

To identify whether the BIM played an important role in the combined treatment strategy in inducing apoptosis, we employed small-interfering RNA targeting BIM in H1975 and PC-9GR cells and subjected to flow cytometric analysis of Annexin V/propidium iodide (PI) staining (Figure 4A, 4B), Our results found that apoptosis was significantly attenuated in combination therapy by BIM knockdown in both cell lines (Figure 4C and 4D).

Next, we examined the relationship between BIM expression level and the clinical outcomes of gefitinib in NSCLC patients. 18 patients with progression-free survival (PFS) of more than 12 months and 18 matched patients with PFS of less than or equal to 3 months (Matching criteria included sex, age, stage and grade) were retrospectively identified (clinical characteristics are shown in Supplementary Table 1). As shown in Figure 4E, significantly higher expression of BIM was found in patients with PFS of more than 12 months.

These data indicated that BIM played an important role as a death regulator in apoptosis induced by the combined treatment with metformin, vorinostat and gefitinib, and the BIM expression might be highly correlated with the prognosis of patients taking gefitinib.

### Metformin inhibited autophagy that was induced by vorinostat and gefitinib cotreatment

**Figure 5:**
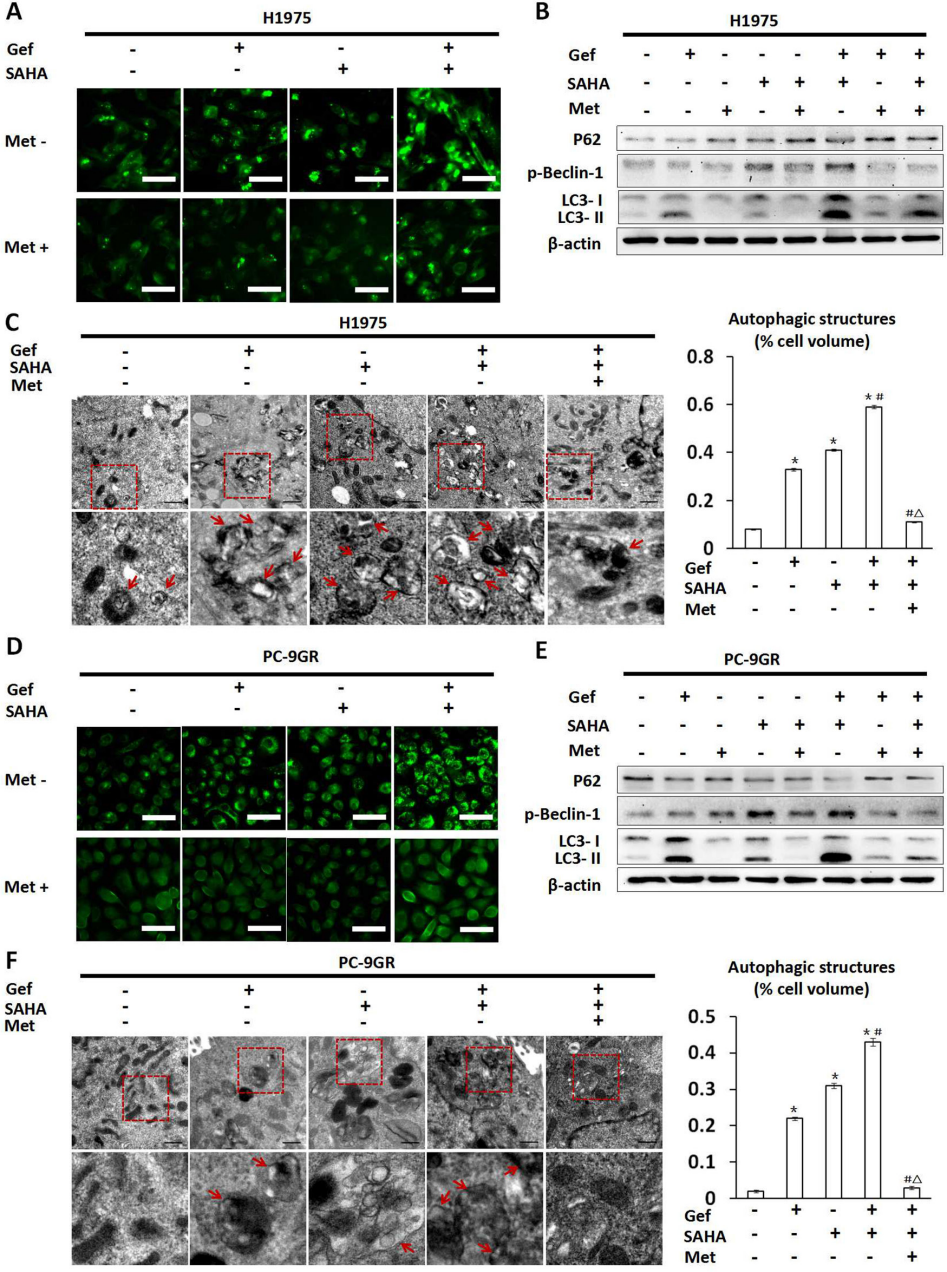
Changes in autophagy seen in H1975 and PC-9GR cells treated with vorinostat and/or gefitinib with/without metformin H1975 and PC-9GR cells were treated with vorinostat (0.5 μM), metformin (1 mM) and gefitinib (IC25) alone or in combination for 48 hr. In **(A)** H1975 and **(D)** PC-9GR cells, fluorescence microscopic imaging showed that vorinostat and gefitinib alone increased punctate fluorescence. In addition, vorinostat and gefitinib cotreatment greatly augmented punctate fluorescence, while metformin reversed increases in punctate fluorescence that were otherwise induced by vorinostat and/or gefitinib treatment. Scale bars: 30μm. **(B)** and **(E)** showed whole cell protein lysates from H1975 and PC-9GR cells under differential treatment were immuno-blotted with antibodies as indicated respectively. The expression of β-actin was used as a loading control. **(C)** and **(F)** Representative autophagic structures and quantitative data obtained by transmission electron microscopy in H1975 and PC-9GR cells, respectively. Scale bars: 0.5μm. Similar results were obtained in three independent experiments. ^*^p < 0.001 as compared with the control (untreated) cells; ^#^*p* < 0.001 as compared with gefitinib or vorinostat treatment alone; Δ*p* < 0.001 as compared with gefitinib combined with vorinostat. Gef: gefitinib; SAHA: vorinostat; Met: metformin.

Given that gefitinib and vorinostat induced autophagy in NSCLC cells [28, 29], and there was a close relationship between autophagy and apoptosis, we next determined whether metformin impacted autophagy that was induced by gefitinib and vorinostat treatment. On autophagic activation, a cytosolic form of LC3 (LC3-I) is cleaved, lipidated, and converted to a conjugated form of LC3 (LC3-II), following which it is inserted into the autophagosome membrane [30]. In H1975 and PC-9GR cells, green fluorescent LC3 dots stained with anti-LC3 antibody were visualized by fluorescence microscopy. In this assay, we found that the combined treatment of vorinostat and gefitinib resulted in a marked increase in LC3 dots when compared with cells that were treated with vorinostat or gefitinib alone (Figure 5A and 5D). That said, when combined with vorinostat, gefitinib induced greater levels of LC3-II conversion (Figure 5B and 5E) when compared with the untreated control as well as under conditions of vorinostat or gefitinib treatment alone. In addition, activation of Beclin-1 and down-regulation of P62 were also considered as autophagic activating factors. The results showed that treatment with either vorinostat alone or vorinostat combined with gefitinib clearly induced the phosphorylation of Beclin-1 in H1975 and PC-9GR cells and decreased P62 levels in PC-9GR cells as compared with untreated controls (Figure 5B and 5E). A transmission electron microscopy (TEM) assay suggested that numerous autophagic vacuoles were appeared in the cells treated with gefitinib or vorinostat. After the combined treatment of gefitinib and vorinostat, the cells exhibited significantly increased autophagic vacuoles, compared with those treated with each agent alone (Figure 5C and 5F).

Conversely, treatment with metformin markedly inhibited autophagy that was induced by vorinostat and gefitinib (Figure 5A and 5D), which was manifested by markedly decreased conversion from LC3-I to LC3-II and phosphorylation of Beclin-1, while increased P62 levels in H1975 and PC-9GR cells (Figure 5B and 5E). TEM results also demonstrated that adding metformin to gefitinib and vorinostat significantly reduced autophagosomes and autophagic structures in H1975 and PC-9GR cells (Figure 5C and 5F).

The results suggested that combined use of gefitinib and vorinostat could enhance autophagy, but lower doses of metformin, when used in an additive capacity, alleviated this effect, which resulted in marked inhibition of autophagy.

### Autophagy inhibition is conducive to enhancing apoptosis induced by combined treatment with vorinostat and gefitinib

**Figure 6:**
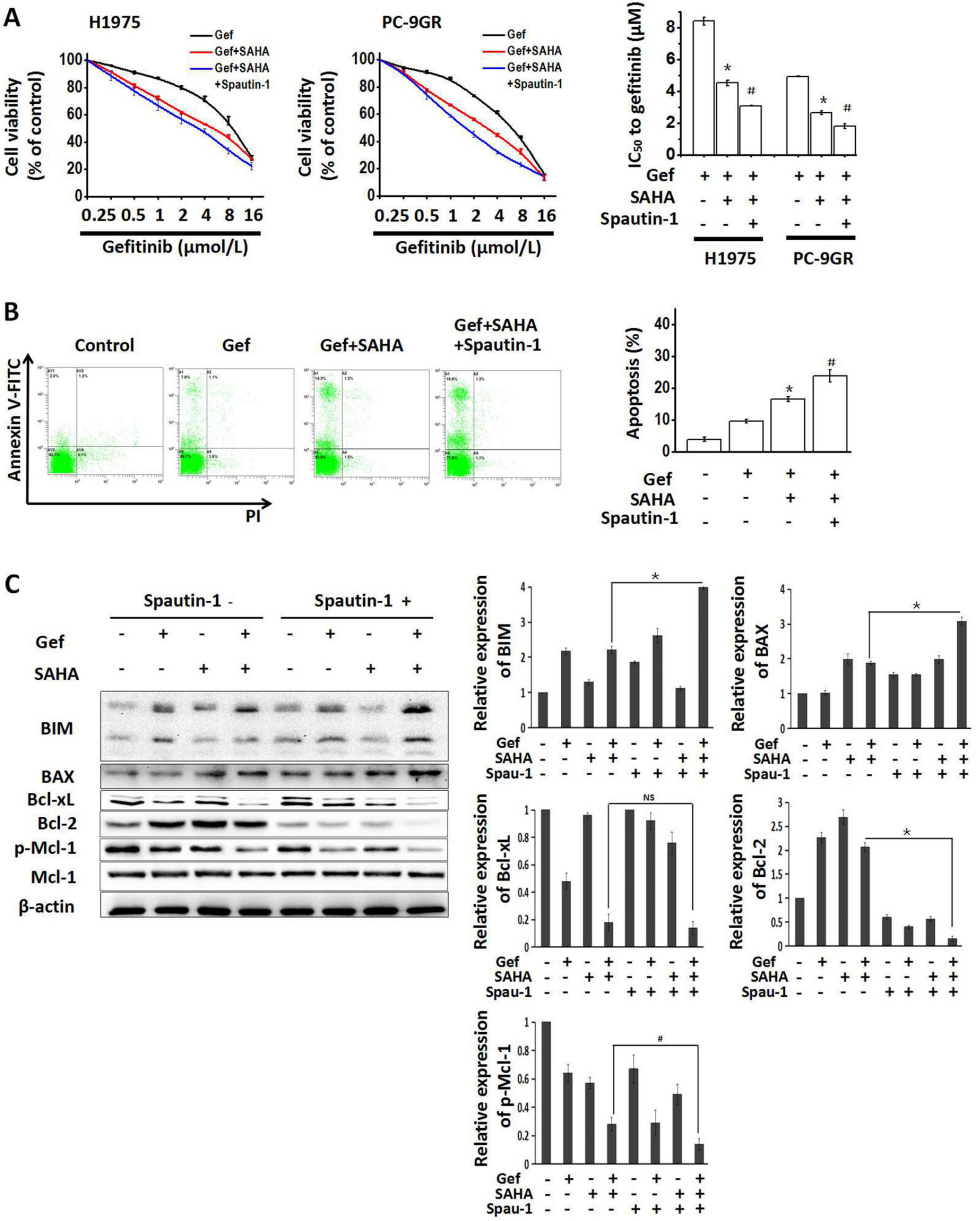
Growth-inhibition and apoptosis-induction by combined treatment with vorinostat and gefitinib were enhanced by inhibiting autophagy **(A)** H1975 and PC-9GR cells were treated with the indicated doses of gefitinib and/or vorinostat (0.5 μM) with/without the potent autophagy inhibitor spautin-1 (10 μM) for 48 hr. The MTT assay was determined growth inhibition effects. ^*^p < 0.05 as compared gefitinib treatment alone; ^#^*p* < 0.05 as compared combined treatment with vorinostat and gefitinib. **(B)** PC-9GR cells were treated with gefitinib alone or combined with vorinostat with/without spautin-1(10 μM) for 48 hr. Flow cytometric analysis observed changes in apoptosis. ^*^p < 0.05 as compared gefitinib treatment alone; ^#^*p* < 0.05 as compared combined treatment with vorinostat and gefitinib. **(C)** Whole cell protein lysates from PC-9GR cells that were treated with gefitinib and/or vorinostat with/without spautin-1 for 48 hr, were immuno-blotted with antibodies as indicated. ^*^p < 0.001; ^#^*p* < 0.05; NS: not significant (Student's t test). Gef: gefitinib; SAHA: vorinostat; Met: metformin.

Since autophagy is closely related to apoptosis [31, 32] and might be involved in the resistance of NSCLC to EGFR-TKI [33], H1975 and PC-9GR cells were treated with the autophagy inhibitor spautin-1 (10 μM) to interfere with the combined effects of vorinostat and gefitinib. As shown in Figure 6A, when combined with vorinostat and gefitinib, spautin-1 significantly potentiated reduced cell viability. Moreover, cotreatment with spautin-1 significantly increased apoptosis that was induced by vorinostat and gefitinib in PC-9GR cells (Figure 6B), In addition, Western blot analyses showed that spautin-1 effectively activated the apoptosis pathway, as seen by upregulated expression of the pro-apoptotic proteins BIM and BAX, decreased Bcl-2 and activated Mcl-1 (Figure 6C). While treatment with spautin-1(10 μM) alone for 48 hr slightly decreased the viability of H1975 and PC-9GR cells (Supplementary Figure 3A and 3B). These findings indicated that enhanced apoptosis induced by combined treatment with vorinostat and gefitinib was partially dependent on inhibiting autophagy in EGFR-TKI-resistant NSCLC cells.

## DISCUSSION

The current study reported for the first time that metformin in combination with vorinostat exerted a synergistic effect in improving the sensitivity of EGFR-TKI resistant NSCLC cells to gefitinib. The effect of this combination against gefitinib resistance was attributed to its ability to induce BIM-dependent apoptosis. Accordingly, we provided the rationale and experimental evidence in support of a combination of vorinostat and metformin in overcoming gefitinib resistance in EGFR-TKI resistant NSCLC patients.

Combination strategies are currently being investigated and have gradually become a focus of attention to overcome EGFR-TKI resistance. However, the approach still requires refinement and optimization in the future [34]. Based on its reliable anti-tumor effects, the roles that pan-HDACis play in adjuvant therapy of NSCLC, particularly in combination with other chemotherapeutic agents and molecular targeted therapies [29, 35, 36] have seen increased attention. While HDACi induce multiple anti-tumor effects including inducing differentiation, inhibiting proliferation and so on, their primary mechanism of anti-tumor activity is via the induction of apoptosis [37]. It has been found that HDAC inhibitors induced apoptosis in particular types of NSCLC cells when combined with irreversible EGFR-TKI or pemetrexed [29, 35], However, the individual tumour cells have heterogenous responses to HDACi induced apoptosis [38], and there’s no significant improvements exit in the overall outcome for advanced NSCLC patients treated with HDACi and erlotinib [13].

In our present study, we also showed that the pan-HDACi vorinostat, in a manner, increased EGFR-TKI resistance NSCLC cells sensitivity to gefitinib. In NSCLC cell lines with different EGFR-TKI resistance mechanism, while gefitinib in combination with vorinostat resulted in significantly decreased cell viability and increased apoptosis, we noted that adding vorinostat could up-regulate the pro-apoptotic proteins BIM and BAX in PC-9GR and H1650-M3 cells but not in H1975 cells, the therapeutic strategy could down-regulate the anti-apoptotic protein Bcl-xL in H1975 cells but not in PC-9GR and H1650-M3 cells. Most of all, it was shown that the addition of vorinostat up-regulated anti-apoptotic protein Bcl-2 in all above three cell lines. These results implied that the NSCLC cells with different EGFR-TKI resistance mechanisms showed a great heterogeneity in the apoptotic response to HDACi. According to relevant literatures [14, 38], it might be inferred that the simultaneous inhibition of Bcl-2, Bcl-xL and Mcl-1 might lead to a significant increase of vorinostat induced apoptosis across diverse NSCLC cell types.

Previous research found vorinostat in combination with Bcl-2 and Bcl-xL inhibitor ABT-263 shows strongly cooperate to overcome heterogeneity associated with response to HDACi across diverse tumour cell types [38]. However, ABT-263 shows no abilities of inhibiting Mcl-1, which is an important factor that affects the effect of this treatment. Meanwhile, in practice, the applications of anti-apoptotic proteins inhibitors are restricted because of its toxicity and high price. Unexpectedly, the anti-diabetic drug metformin is an effective, economic and safe assistance drug for cancer, which can enhance cellular sensitivity to EGFR-TKI [21, 22] and may synergistically enhance the anti-tumor activity of HDACis [39]. More important, metformin can inhibit the expression of anti-apoptotic proteins such as Bcl-2 and Mcl-1 [23], In the present study, and as expected, metformin could effectively overcome the deficiency of vorinostat in regulating anti-apoptotic Bcl-2 family proteins, the addition of low-dose metformin could significantly inhibit Bcl-2, Bcl-xL and Mcl-1, and further enhanced the expression levels of BIM and BAX, and synergistically induced apoptosis and enhanced the sensitivity of gefitinib in these NSCLC cells. The results suggested that the combination of vorinostat and metformin could formally represent a novel strategy to overcome EGFR-TKI resistance across diverse NSCLC cells displaying different resistance mechanisms. Furthermore, the clinical dosage in this combination strategy was much lower than was previously reported [21, 29]; hence, it is indicated that this combination strategy may display a dampened side-effect profile while displaying a higher potency.

Reportedly, the pro-apoptosis protein BIM is closely related to EGFR-TKI resistance [40], and is an important target of HDACi [11, 12], Based on the observations, we further demonstrated that the combination treatment was dependent on BIM and that silencing of BIM expression resulted in greatly reduced apoptosis and cell death that was induced by the combination treatment. These findings demonstrated that increased BIM levels played a major role in the synergistic cytotoxic effect of vorinostat when combined with metformin.

The role of autophagy in cancer treatment is complex and it is unclear under which conditions autophagy promotes cell death or cell survival. Studies have reported that inhibition of autophagy could overcome erlotinib resistance by inducing apoptosis and triggering ER stress [19], suggesting the requirement for autophagy inhibition to enhance the effect of gefitinib. In this study, we found that both vorinostat and gefitinib elicited robust induction of autophagy in H1975 and PC-9GR cells, which was siginificantly inhibited by metformin. Next, we confirmed the synergistic effect of inhibited autophagy under conditions of combinatorial treatment with vorinostat, gefitinib and spautin-1, showed that spautin-1 could significantly increase BIM expression level and apoptosis induction by combined use of gefitinib and vorinostat. These observations indicated that the inhibition of gefitinib and vorinostat-induced autophagy might have played a role in the induction of apoptosis, and metformin might as well contribute to the effect of apoptosis inducing by inhibiting autophagy.

Taken together, these findings suggested that treatment with metformin coupled with vorinostat significantly improved EGFR-TKI sensitivity in NSCLC cells that exhibit intrinsic or aquired EGFR-TKI resistance, and did so by inducing BIM-dependent apoptosis.

## MATERIALS AND METHODS

### Cell-lines and reagents

The gefitinib-sensitive PC-9 cells (harboring a deletion in exon 19 of EGFR) were gifted by Prof. J. Xu and Dr. M. Liu from Guangzhou Medical University (China). We generated resistant clones of PC-9 cells by chronic exposing the cells to increasing concentrations of gefitinib, as described previously [41]. The concentration of gefitinib was increased stepwise (from 0.02μM to 2μM) when the cells resumed proliferation similar to the pattern in untreated parental cells. The resultant PC-9GR cells were resistant to gefitinib *in vitro* (Supplementary Figure 1). H1975 cells were obtained from the American Type Culture Collection (ATCC, Manassas, VA, USA), which harbour L858R and T790M mutations in the EGFR gene and are resistance to gefitinib [42]. H1650-M3 cells were a kind gift from Dr. R. Sordella from the Cold Spring Harbor Laboratory, which harbour an exon 19 in-frame deletion (delE746-A750) of the EGFR gene and homozygous deletion of PTEN, and are associated with gefitinib resistance [42]. These cells were cultured in RPMI-1640 medium (HyClone) containing 10% FBS (Gibco), 2 mmol/L L-glutamine solution (Gibco), 100 μg/mL streptomycin (HyClone), and 100 U/mL penicillin (HyClone) at 37°C in an atmosphere of 5% CO2 in air and a fully humidified (90% humidity) incubator.

Gefitinib (Iressa) was obtained from Tocris Bioscience and stored as 10 mmol/L stock solutions in dimethyl sulfoxide (DMSO) at -20°C. Metformin (Sigma) was stored as 1 mol/L stock solutions in deionized water at -20°C. Vorinostat (Sigma) and spautin-1 (Selleck) were dissolved in DMSO and stored at -20°C.

### Cell growth and invasion assays

The cytotoxic effects of gefitinib plus vorinostat and/or metformin were determined by the MTT dye reduction method and BrdU incorporation assay [43]. CI was calculated by the CompuSyn software program (ComboSyn, Inc., Paramus, NJ, USA), and assuming 1 as the cutoff. A CI<1 represented a synergistic effect, a CI = 1 represented an addictive effect and a CI>1 indicated an antagonistic effect [44]. Cell invasion was measured with a 24-well specific 6.5-mm-diameter inserts (8.0-μm pore size; Corning Incorporated). The relative cell invasion index was calculated as previously reported [45].

### Flow cytometry assays

Cellular apoptosis were detected by flow cytometric analysis. Cells were collected at 48 hr after different treatments by trypsinization, and then washed three times with phosphate-buffered saline (PBS), and resuspended at a density of 1 × 107 cells/mL. After double staining using the FITC Annexin V Apoptosis Detection Kit I and PI for 30 min at ambient temperature in the dark, cells were analyzed using a flow cytometer (Beckman Coulter Novios, FL, USA).

### RNA interference

Cells were cultured in 6-well plates with 3 × 106 cells per well. After 24 hr, the cells were transfected with small interfering RNA (siRNA) against BIM using Hiper-Fect Transfection Reagent (QIAGEN, Hilden, Germany) in accordance with the manufacturer’s instructions.

The siRNA target sequences were as follows:

BIM negative control: 5′-UUCUCCGAACGUGUCACGUdTdT-3′and 5′-ACGUGACACGUUCGGAGAAdTdT-3′
BIM # 1: 5′- GCCACAAGGUAAUCCUGAAdTdT-3′and 5′- UUCAGGAUUACCUUGUGGCdTdT-3′
BIM # 2: 5′-GACGAGUUUAACGCUUACUdTdT-3′ and 5′- AGUAAGCGUUAAACUCGUCdTdT -3′
BIM # 3: 5′- GAGACGAGUUUAACGCUUAdTdT-3′and 5′-UAAGCGUUAAACUCGUCUCdTdT-3′ (GE Dharmacon). Cells were harvested 48 hr post-transfection.

### Western blotting

Cells were harvested and lysed in a lysis buffer that was supplemented with protease and phosphatase inhibitors. After 3 min of lysis, the cell debris was removed by centrifugation at 12,000 × g for 30 min, and the protein concentration was determined by the Bradford method (Millipore, Billerica, MA, USA). Next, equal quantities of protein were separated by a 12% SDS-PAGE and then transferred to PVDF membrane (Millipore, Billerica, MA, USA) using an iBlot™ dry blotting system (BIO-RAD). Then, after blocking the membrane with 5% skim milk proteins, the membranes were cross-reacted with specific antibody at 4^°^C overnight (at 1:1000 dilution). The membranes were subsequently washed in TRIS-buffered saline/tween-20 (TBST) and incubated with horseradish peroxidase-conjugated secondary antibody for 1 hr (at 1:5000 dilution), following which, specific protein bands on the membranes were observed using an ECL detection system (BIO-RAD). The primary antibodies and the secondary antibodies (HRP-conjugated anti-rabbit IgG) were obtained from Cell Signaling Technology. The control for equal protein loading was assessed with an anti-β-actin antibody (Bioss).

### Cyto-ID autophagy detection

Cells were seeded in 6-well plates at a density of 3 × 106 cells per well and then treated with gefitinib (IC25) alone or co-treated with vorinostat (0.5μM) and/or metformin (1mM) for 48 hr. Next, the cells were washed three times with PBS and were then incubated in Cyto-ID (1 μl Cyto-ID/1ml PBS) for 15 min according to the manufacturer’s instructions (Cyto-ID® Autophagy Detection Kit, ENZ-51031-K200, ENZO, Farmingdale, NY, USA). Next, cells were washed twice with PBS and observed by confocal microscopy.

### Transmission electron microscopy

Cell samples were fixed overnight in 2% glutaraldehyde and at 4°C and washed in 0.05 sodium cacodylate buffer, postfixed in 1% osmium tetroxide, dehydrated in graded ethanol and propylene oxide. Ultrathin sections were prepared, collected on copper grids, and were stained with lead citrate and 0.5% uranyl acetate, and examined with TEM (Philips TECNAI 10) installed in the Biomedical Analysis Center at Third Military Medical University. Cell autophagy was quantified by measuring the volume occupied by autophagic structures, according to the guidelines [46].

### Patients and specimens

Specimens were collected from patients with advanced NSCLC and activating EGFR mutations, all patients had already accepted gefitinib as first-line treatment between January 2013 and December 2015 at Daping Hospital of the Third Military Medical University (Chongqing, China). All the tissue section slides were baked at 60°C for 2hr, followed by deparaffinized and hydrated, and incubated with 3% hydrogen peroxide. After being microwaved in 0.01M sodium citrate buffer for 30min, the sections were preincubated in 10% normal goat serum for 30 min to prevent nonspecific staining. Subsequently, the sections were incubated with the BIM polyclonal antibody overnight at 4°C. Sections were then sequentially incubated with biotinylated goat anti-rabbit secondary antibody (Dako, Glostrup, Denmark) for 30 min each at room temperature. The immunostaining results were substantiated independently by two experienced pathologists blinded to the patient’s identity and clinical status. Scoring for IHC staining was based on the criteria described previously [47]. The study was approved by the Ethics Committee of Daping Hospital of the Third Military Medical University. Informed consent was obtained from all individual patients or relatives included in the study.

### Statistical analysis

All data were expressed as mean ± SEM of triplicate samples and analyzed using student’s t test. Statistical analyses were performed using the SPSS v.17.0 statistical software package (SPSS Inc., Chicago, IL, USA). Statistical significance was assumed at an alpha value of P <0.05.

## SUPPLEMENTARY MATERIALS FIGURES AND TABLE



## Abbreviations

EGFR-TKI: epidermal growth factor receptor tyrosine kinase inhibitor
HDACi: histone deacetylase inhibitor
NSCLC: non-small cell lung cancer
Bcl-2: B-cell lymphoma 2
CI: combination index
PFS: progression-free survival
TEM: transmission electron microscopy
PBS: phosphate-buffered saline
PI: propidium iodide
siRNA: small interfering RNA
TBST: TRIS-buffered saline/tween-20
