# Supplementary material for: Vorinostat and metformin sensitize EGFR-TKI resistant NSCLC cells via BIM-dependent apoptosis induction
Source: Oncotarget. 2017 Sep 23;8(55):93825–38. doi: 10.18632/oncotarget.21225 (PMC5706838; doi:10.18632/oncotarget.21225)
Supplement: Supplementary file 1 [file oncotarget-08-93825-s001.pdf]

Vorinostat and metformin sensitize EGFR-TKI resistant NSCLC cells via BIM-dependent apoptosis induction

SUPPLYMENTARY MATERIALS

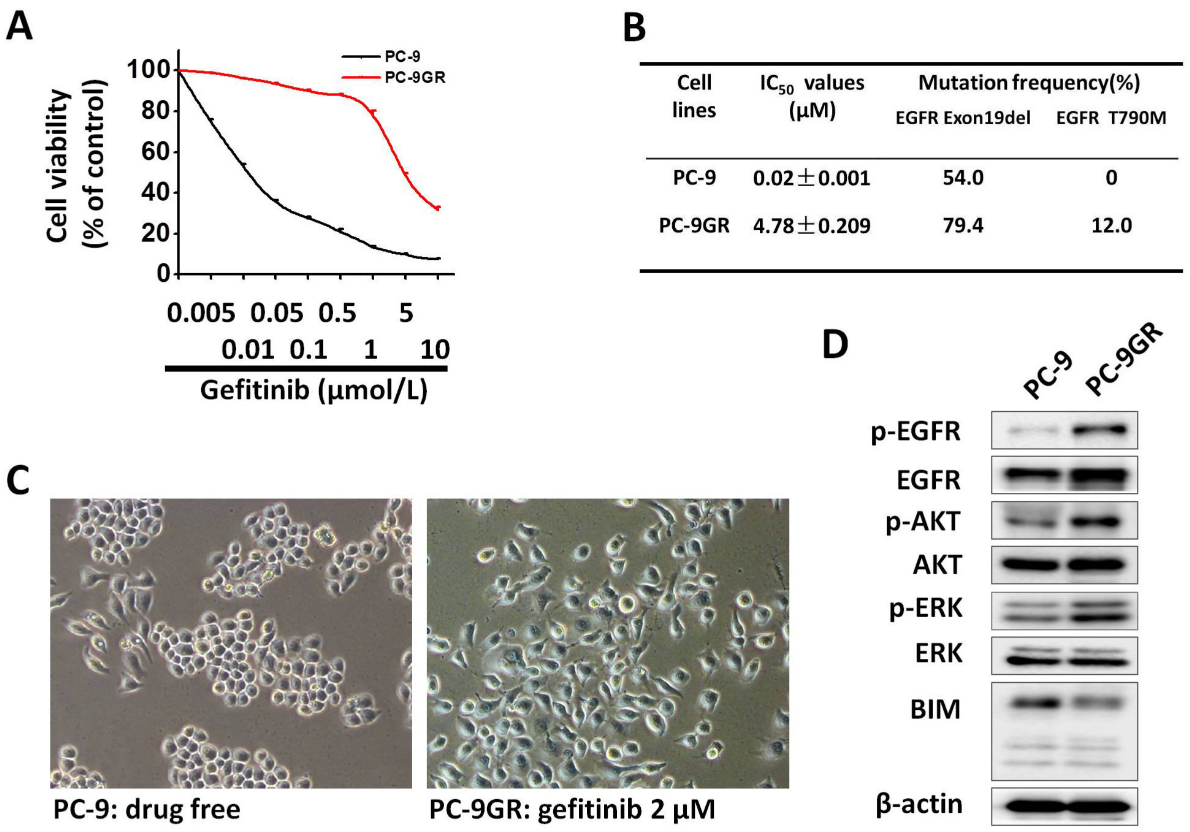

Supplementary Figure 1: Establishment of PC-9GR cells by chronic, repeated exposure to increasing concentrations of gefitinib. **(A)** The PC-9 cell line was made resistant to gefitinib by exposuring it in increasing concentrations of gefitinib. Parental and resistant PC-9GR cells were treated with gefitinib at the indicated concentrations for 48hr. Cell viabilities of PC-9, PC-9GR were assessed by the MTT assay. **(B)** whole-exomesequencing confirmed the presence of the T790M mutation in gefitinib-resistant PC-9GR cells. **(C)** Morphological differences observed between PC-9 and PC-9GR cells. **(D)** Western blotting analysis was performed using indicated protein-specific antibodies.

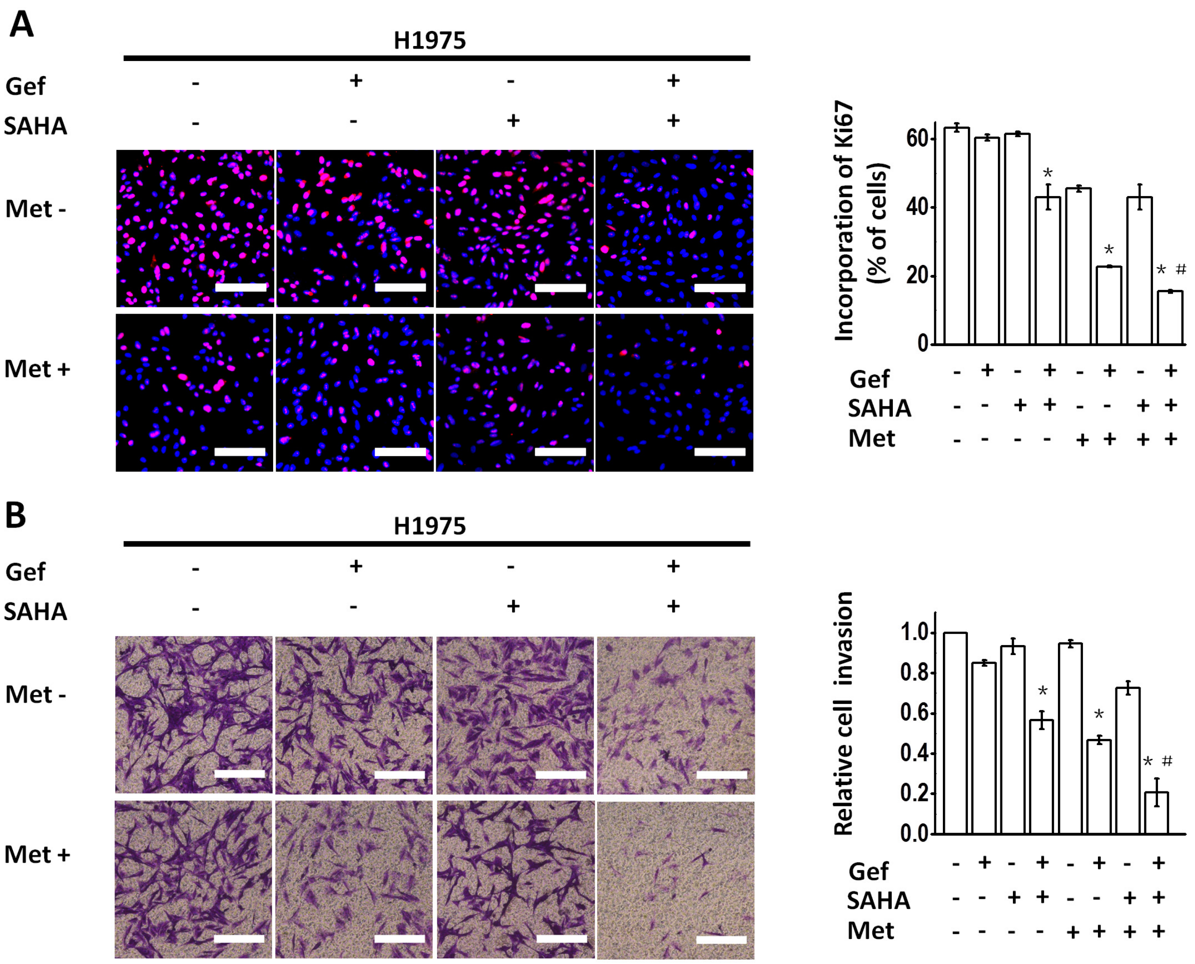

Supplementary Figure 2: Metformin and vorinostat synergistic increased gefitinib sensitivity in H1975 cells. (A) Analysis of cell proliferation by the BrdU incorporation assay in H1975 cells that were treated with vorinostat (0.5 μM), metformin (1 mM) and gefitinib (IC25) alone or when combined for 48 hr. Scale bars: 100 μm. **(B)** Analysis of cell invasiveness by the cell invasion assay in H1975 cells that were treated with vorinostat (0.5 μM), metformin (1 mM) and gefitinib (IC25) when used alone or in combination for 48 hr. Scale bars: 100 μm. $^{*}p < 0.05$ as compared with gefitinib treatment alone; $^{\#}p < 0.05$ as compared with a combined treatment treatment with vorinostat or metformin and gefitinib. Gef: gefitinib; SAHA: vorinostat; Met: metformin.

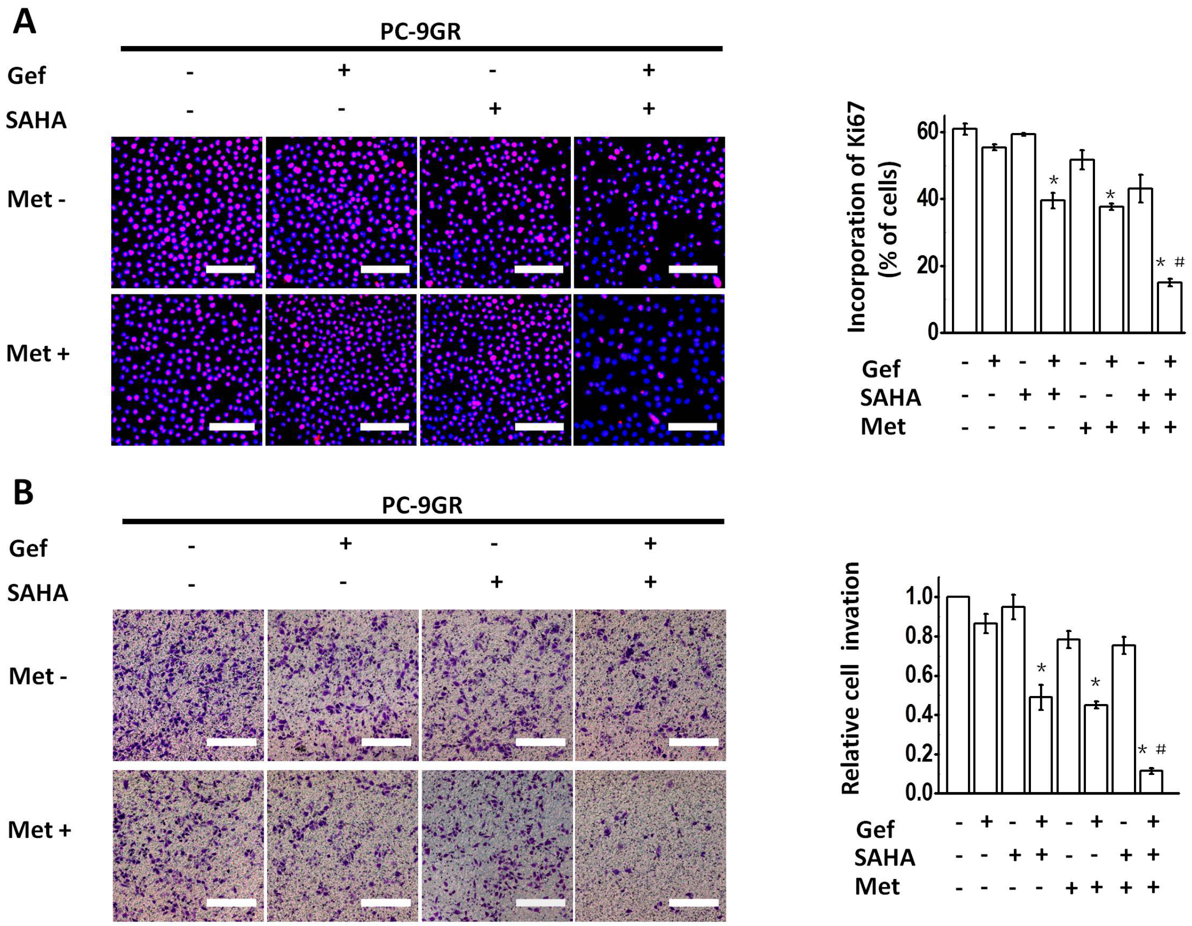

Supplementary Figure 3: Metformin and vorinostat synergistic increased gefitinib sensitivity in PC-9GR cells. (A) Analysis of cell proliferation by the BrdU incorporation assay in PC-9GR cells that were treated with vorinostat (0.5 μM), metformin (1 mM) and gefitinib (IC25) alone or when combined for 48 hr. Scale bars: 100 μm. **(B)** Analysis of cell invasion by the cell invasion assay in PC-9GR cells that were treated with vorinostat (0.5 μM), metformin (1 mM) and gefitinib (IC25) alone or in combination for 48 hr. Scale bars: 100 μm. $^{*}p < 0.05$ as compared with gefitinib treatment alone; $^{\#}p < 0.05$ as compared with combined treatment with vorinostat or metformin and gefitinib. Gef: gefitinib; SAHA: vorinostat; Met: metformin.

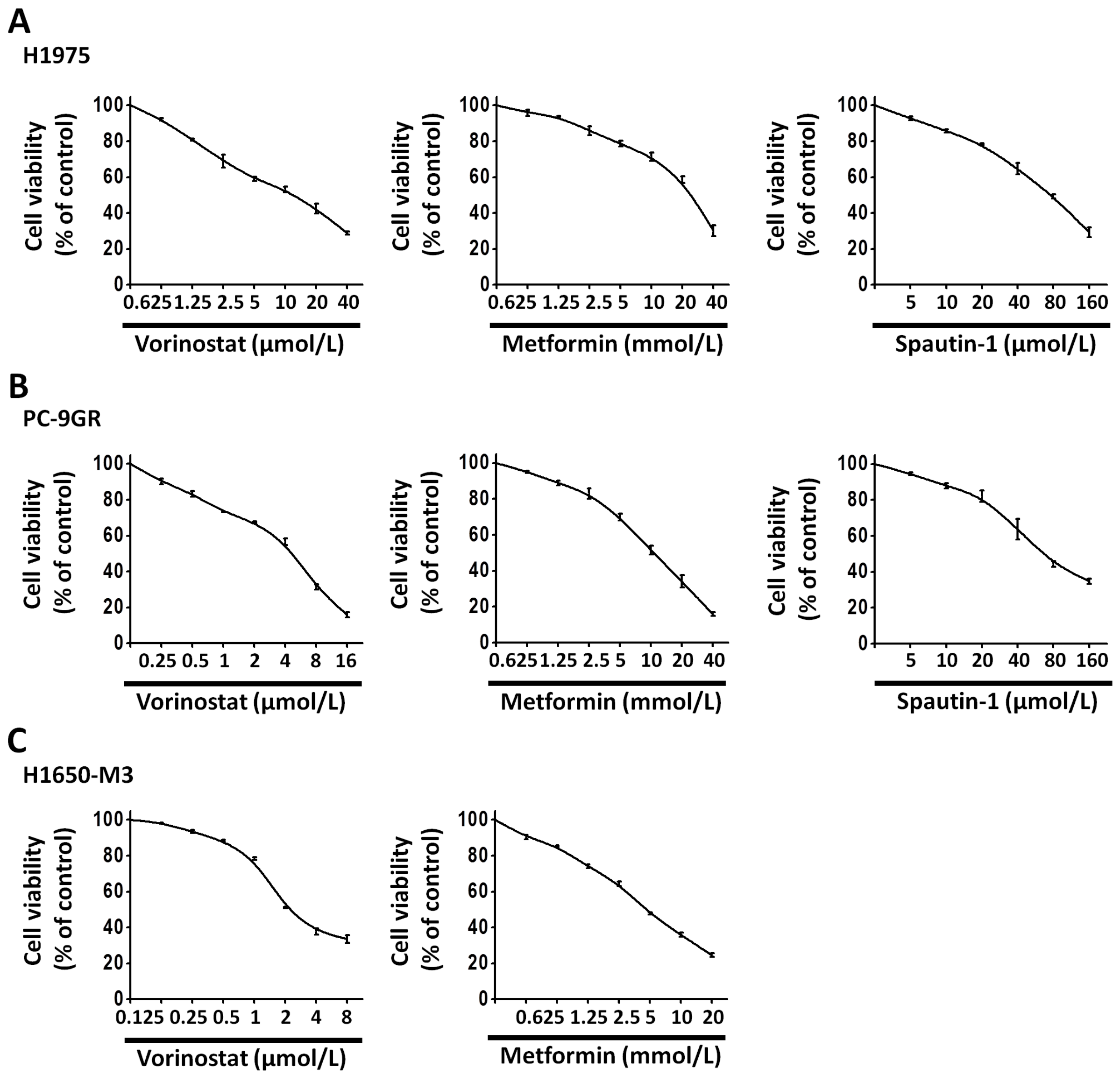

Supplementary Figure 4: Cell growth curves as determined by MTT test. **(A)** H1975, **(B)** PC-9GR and **(C)** H1650-M3 cells were dose-dependently treated with vorinostat, metformin or spautin-1 for 48 hr and cell viability was measured by MTT assay.

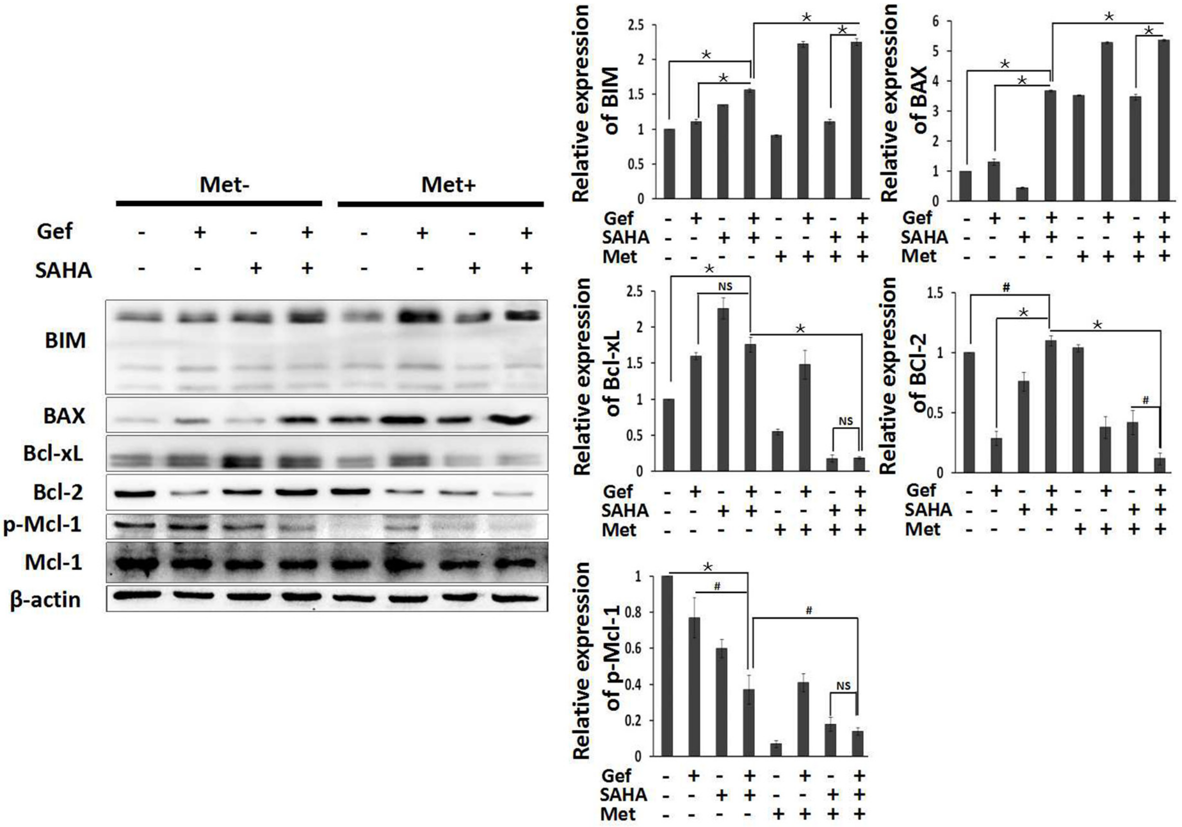

Supplementary Figure 5: Metformin in combination with vorinostat and gefitinib regulated apoptosis signal pathway in H1650-M3 cells. H1650-M3 cells were treated with vorinostat (0.5 μM) and/or gefitinib (IC25) with/without metformin (1 mM) for 48 hr. Whole cell protein lysates were immunoblotted and detected by the indicated protein-specific antibodies. $^{*}p < 0.001$; $^{\#}p < 0.05$; NS: not significant (Student's t test). Gef: gefitinib; SAHA: vorinostat; Met: metformin. Gef: gefitinib; SAHA: vorinostat; Met: metformin.

Supplementary Table 1: Characteristics of 36 NSCLC patients with activating EGFR mutations

Patients characteristics

Variables	Total no. (%)	PFS>12months [no.(%)]	PFS≤3months [no.(%)]	*P* Value
**Age(years)**				
≤60	21	11	10	0.735
>60	15	7	8	
**Sex**				
Male	10	5	5	1
Female	26	13	13	
**Tobacco smoking**				
Yes	9	5	5	0.328
No	21	12	9	
Unknown	6	1	4	
**TNM stage**				
IIIB	13	8	5	0.298
IV	23	10	13	
**Histology grade**				
Well	15	8	7	0.347
Moderate	19	10	9	
Poor and Unknown	2	0	2	
**EGFR mutation status**				
Exon 21 L858R mutation	14	6	8	0.494
Exon 19 deletion	22	12	10	
